# Cross-Linked Chitosan/Multi-Walled Carbon Nanotubes Composite as Ecofriendly Biocatalyst for Synthesis of Some Novel Benzil Bis-Thiazoles

**DOI:** 10.3390/polym13111728

**Published:** 2021-05-25

**Authors:** Latifah A. Alshabanah, Sobhi M. Gomha, Laila A. Al-Mutabagani, Tariq Z. Abolibda, Nahed A. Abd El-Ghany, Waleed A. M. A. El-Enany, Ahmed K. El-Ziaty, Rania S. Ali, Nadia A. Mohamed

**Affiliations:** 1 Department of Chemistry, College of Science, Princess Nourah bint Abdulrahman University, Riyadh 11671, Saudi Arabia; laalsabanah@pnu.eud.sa; 2 Department of Chemistry, Faculty of Science, Cairo University, Giza 12613, Egypt; abdelghanyn@sci.cu.edu.eg (N.A.A.E.-G.); waleed_org_chem@yahoo.com (W.A.M.A.E.-E.); namadm@hotmail.com (N.A.M.); 3 Department of Chemistry, Faculty of Science, Islamic University in Almadinah Almonawara, Almadinah Almonawara 42351, Saudi Arabia; t.z.a@iu.edu.sa; 4 Chemistry Department, Faculty of Science, Ain Shams University, El-Khalifa El-Mamoun, Abbassia, Cairo 11566, Egypt; ahm512@sci.asu.edu.eg; 5 Department of Basic Science, Faculty of Technology and Education, Helwan University, Cairo 11795, Egypt; raniayousif2016@yahoo.com; 6 Department of Chemistry, College of Science & Arts, Qassim University, Buraidah 51452, Saudi Arabia

**Keywords:** thiosemicarazones, hydrazonoyl halides, bis-thiazoles, cross-linked chitosan, multiwalled carbon nanotubes/cross-linked chitosan composite

## Abstract

Aminohydrazide cross-linked chitosan (CLCS) and its MWCNTs (CLCS/MWCNTs) were formulated and utilized as a potent ecofriendly basic heterogeneous biocatalyst under ultrasonic irradiation for synthesis of two novel series of benzil bis-aryldiazenylthiazoles and benzil bis-arylhydrazonothiazolones from the reaction of benzil bis-thiosemicarbazone with 2-oxo-*N*′-arylpropanehydrazonoyl chlorides and ethyl 2-chloro-2-(2-phenylhydrazono) acetates, respectively. The chemical structures of the newly synthesized derivatives were elucidated by spectral data and alternative methods, where available. Additionally, their yield % was estimated using a traditional catalyst as TEA and green recyclable catalysts as CLCS and CLCS/MWCNTs composite in a comparative study. We observed that, under the same reaction conditions, the yield % of the desired products increased by changing TEA to CLCS then to CLCS/MWCNT from 72–78% to 79–83% to 84–87%, respectively. The thermal stability of the investigated samples could be arranged as CLCS/MWCNTs composite > CLCS > chitosan, where the weight losses of chitosan, CLCS and CLCS/MWCNTs composite at 500 °C were 65.46%, 57.95% and 53.29%, respectively.

## 1. Introduction

In recent decades, much research has focused on green chemistry or environmentally benign chemistry to reduce the risk to humans, minimize environmental pollution, decrease the hazardous impacts of chemicals and eliminate wastes. A green chemical reaction ordinarily uses renewable and regenerable materials as a catalyst. In this respect, the importance of environment-friendly resources and procedures is increasing, as is the use of natural materials in catalysis instead of the transition metals which lead to environmental pollution [1]. Biopolymers such as cellulose, starch and chitosan are a class of materials that are cheap, eco-friendly and widely available in nature [2]. In recent years, chitosan as a biopolymer attracted much attention as an active catalyst in organic chemical reactions due to its unique properties of biocompatibility, biodegradability and antimicrobial activity [3,4,5]. 

In organic synthesis, the catalyst is a vital factor to minimize the reaction time and to obtain the desired product in high yield. Homogenous catalysts provide some benefits, such as fast dissolution rate, radially accessible active sites for substrates, tracking real-life mechanistic analysis and strong specificity to target products [6]. Heterogeneous catalysts have many advantages over homogeneous catalysts in chemical reactions, such as easy removal, recovery, recycling and the ability to obtain the pure products with minimal involvement in purification steps. In this respect, chitosan is considered an efficient heterogeneous catalyst or a catalyst support [7]. Chitosan can play an important role as an abundant, bioactive, biocompatible, biodegradable and renewable green material. It is derived from chitin, the main component of crustacean shells, by alkaline hydrolysis. Chitosan is a hydrophilic molecule that has basic amino groups available for binding with different metal ions which allows easy functionalization and creation of new desired properties [7,8,9,10,11]. Chitosan is commonly used as a green and renewable polymeric catalyst in a wide range of reactions. The chitosan-based catalysts are not only the most active and selective in catalytic reactions, but they are also promising in organic catalysis due to their “green” accessibility [12]. Furthermore, chitosan can easily be modified chemically or physically to become a versatile catalytic material in organic transformation. The reusable and recycle nature of chitosan-based organocatalysts provides advantages over traditional and conventional catalysts [13]. The cross-linking of chitosan is a useful method to increase thermal stability, mechanical strength and chemical resistance, and to produce porous chitosan. Porosity of solid catalysts is essential for acceptable catalyst activities, since high porosity and high surface area allow the easy access of reactant molecules, thus enhancing the chemical reaction [14,15]. With the advancement of nanotechnologies, owing to their nano-size and large surface area chitosan nanocomposites have attracted wide attention. Recently, carbon nanotubes (CNTs) and chitosan composites have been researched to combine the interesting properties of CNTs and chitosan [16,17,18,19]. CNTs are characterized by their light weight and high tensile strength with excellent chemical and thermal stability and therefore can enhance the mechanical properties of polymers. Thus, incorporation of CNTs into chitosan matrices resulted in nanocomposites with superior mechanical properties and large surface areas, enhancing catalytic materials [20].

On the other hand, bis-thiazoles are known for their remarkable biological activities, having anticancerous, antibiotic, antimicrobial, antifungal, antibacterial, tuberculostatic, antiamoebial and anti-inflammatory activities [21,22,23,24,25,26,27].

In the present work, we have prepared a novel series of bis-thiazoles from reaction of benzil bis-thiosemicarbazone with the respective hydrazonoyl halides in high-percentage yield using a chemically cross-linked chitosan/MWCNTs composite as a basic catalyst. Cross-linking chitosan with an unconventional cross-linker (*p*-aminobenzhydrazide) imparts many advantages to chitosan, as it increases the functional groups (hydroxyl, amino, hydrazide and phenyl groups) in the chitosan matrix, thus increasing the active sites available to the reactants. Additionally, it is easily separated and recovered from the reaction mixture due to its insolubility in all organic solvents. Moreover, it can be reused several times without reduction in performance. Furthermore, incorporation of MWCNTs into cross-linked chitosan matrix resulted in an increase in its available surface area and improvement of its thermal properties. Thus, cross-linked chitosan/MWCNTs composite can efficiently be used as a catalyst in both chemical synthesis and environmental remediation areas.

## 2. Results and Discussion

### 2.1. Preparation of CLCS and CLCS/MWCNTs Composite

**Figure 1:**
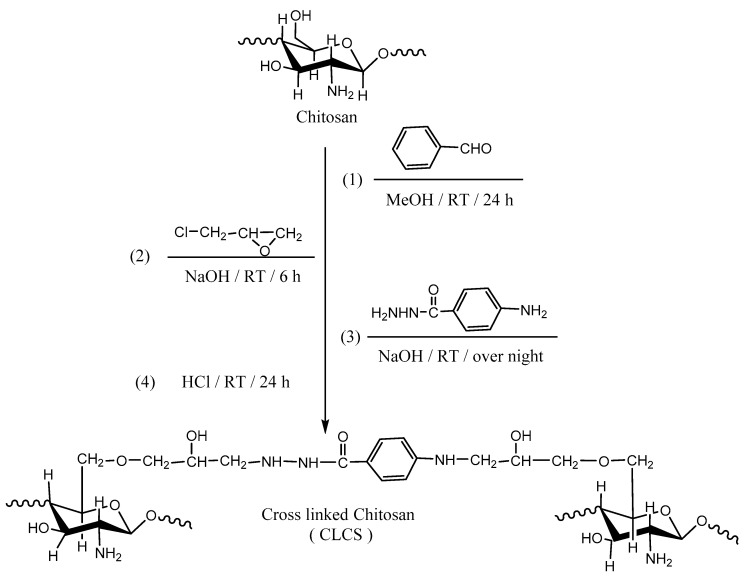
Schematic representation of CLCS preparation.

**Figure 2:**
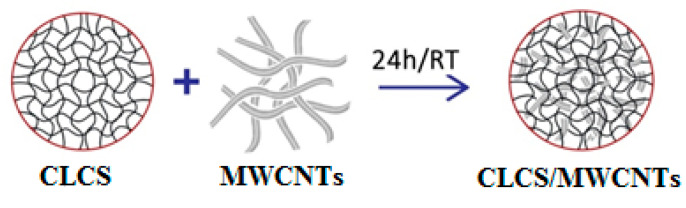
Schematic representation of CLCS/MWCNTs preparation.

Chitosan as a natural polymer is considered to be one of the green raw materials suitable for industrial and medical applications because of its wonderful properties, such as superior ability for bio-degradation and bio-compatibilization and its nontoxicity. Cross-linking of chitosan takes place to introduce additional functionality to its structure that can expand the fields of its applications. Here, CLCS and a CLCS/MWCNTs composite were prepared according to our recently published method [28] via a four-step procedure, during which the primary amine groups in chitosan were firstly protected by reaction with benzaldehyde to produce chitosan Schiff’s base, in which the primary hydroxyl groups on C6 were reacted with epichlorohydrine. Then, the resulting epoxy rings were reacted with 4-aminobenzhydrazide and, finally, the obtained aminohydrazide cross-linked chitosan Schiff’s base was hydrolized in acidic medium to eliminate the benzaldehyde moieties and retrieve the amino groups in the produced CLCS. The procedure steps are depicted in Figure 1. Further modification of CLCS was preceded by implanting MWCNTs inside its matrix to produce a CLCS/MWCNTs composite as represented in Figure 2. 

### 2.2. FTIR Spectra of CLCS and CLCS/MWCNTs Composite

**Figure 3:**
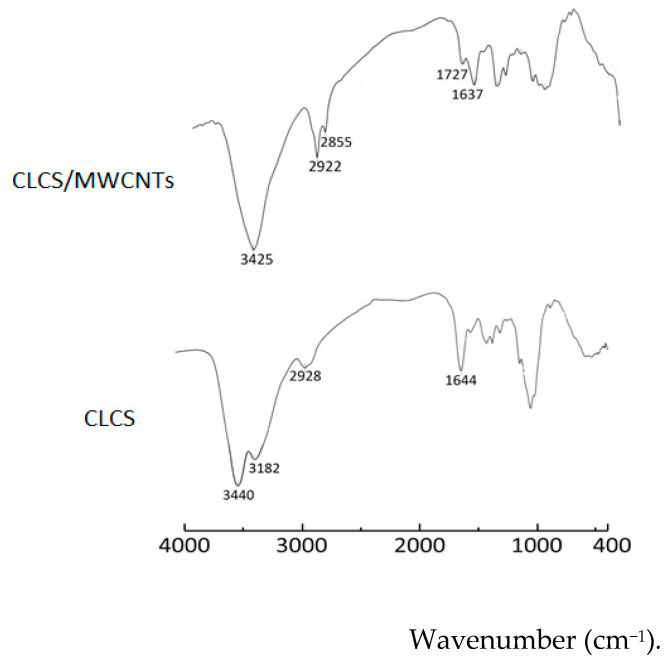
The FTIR spectra of CLCS and CLCS/MWCNTs composite.

The FTIR spectra of CLCS and its MWCNTs composite are shown in Figure 3. The observed spectra showed that the ionic interaction between the pi-bonds of MWCNT sidewalls and CLCS functional groups resulted in a change in the location of several absorption bands to lower frequencies, as well as the emergence of new absorption bands. The absence of the doublet peaks of CLCS’s free amino groups at 3440 and 3182 cm^−1^, as well as the presence of a single broad band that moved to lower frequencies at 3425 cm^−1^ for the CLCS/MWCNTs composite, indicate that these groups are involved in interactions with MWCNTs. The new peaks in the CLCS/MWCNTs composite at 2855 cm^−1^ may be attributed to the MWCNTs integrated into the CLCS matrix. Furthermore, the new peak seen in the CLCS/MWCNTs composite at 1727 cm^−1^ could be due to the free CO group of the amide I and the hydrazide moiety, suggesting nearly complete hydrogen bond cleavage. In the CLCS/MWCNTs composite, the peak at 1644 cm^−1^ relative to the overlapped amide I and CO of the hydrazide moiety moved to 1637 cm^−1^. This may be due to their overlapping with the incorporated MWCNTs’ C=C vibration frequency. These peaks confirm the nanocomposite’s creation. 

### 2.3. Scanning Electron Microscopy (SEM) of CLCS and CLCS/MWCNTs Composite

**Figure 4:**
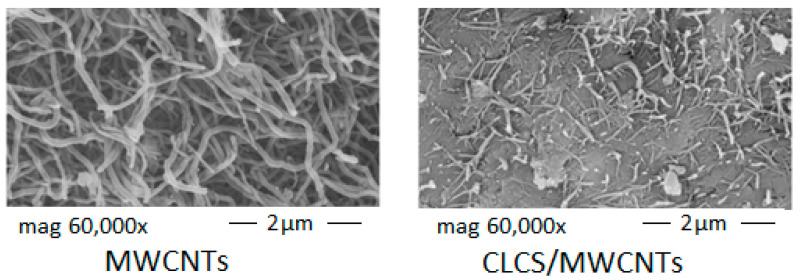
SEM images of MWCNTs and CLCS/MWCNTs composite at a magnification of 60,000×.

Figure 4 shows the surface morphology of the CLCS/MWCNTs composite observed using scanning electron microscopy. It appears from the image that MWCNTs are equally dispersed onto the surface of the CLCS matrix. 

### 2.4. X-ray Diffraction of CLCS and CLCS/MWCNTs Composite

**Figure 5:**
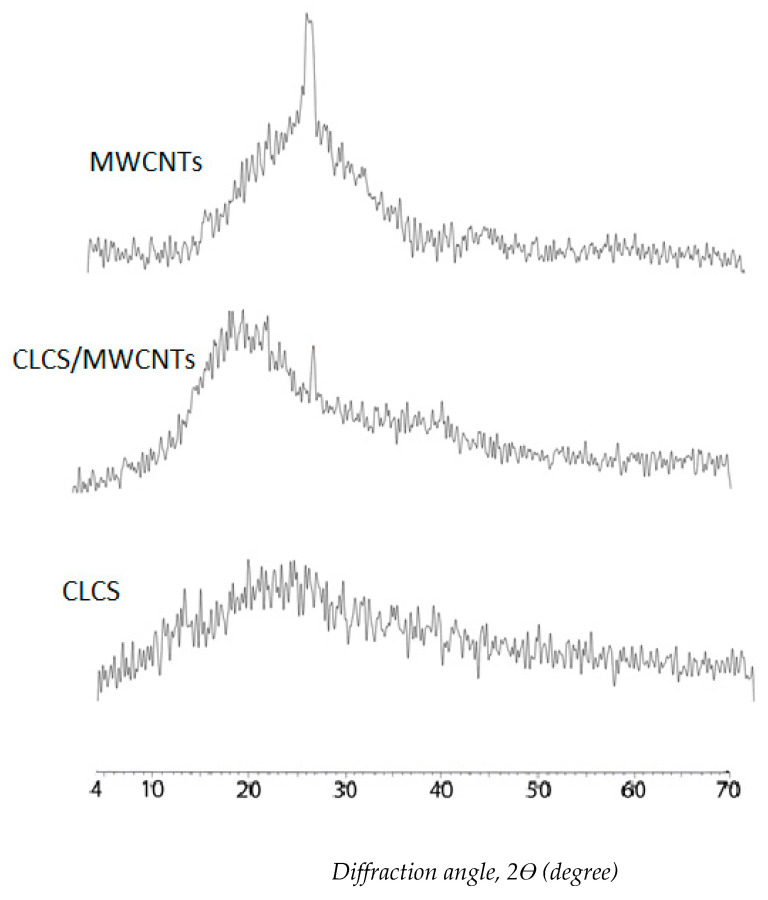
X-ray diffraction patterns of CLCS, CLCS/MWCNTs composite and MWCNTs.

The inner structure of the nanocomposite was studied using the X-ray diffraction technique. The patterns revealed that MWCNTs alone have a crystalline and ordered structure, as shown by the peak at 2 = 26.8°, while free CLCS has an amorphous structure (Figure 5). The presence of a new peak around 2 = 20° in the nanocomposite pattern, which is related to MWCNTs, confirms the incorporation of MWCNTs into CLCS. As a result, it appears that MWCNTs diffused within the CLCS matrix, filling the matrix’s inner spaces (cavities), resulting in the MWCNTs/CLCS composite.

### 2.5. Thermal Stability of CLCS and CLCS/MWCNTs Composite

**Figure 6:**
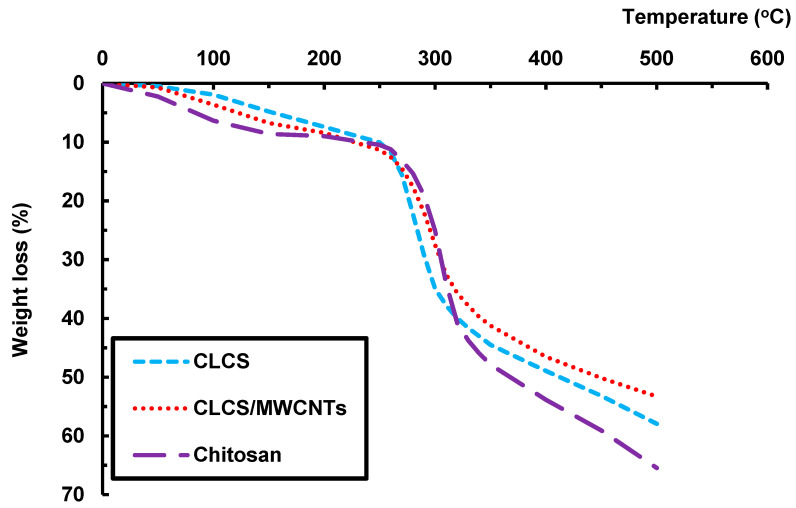
Typical TG thermograms of chitosan, CLCS and CLCS/MWCNTs composite. All the thermograms were recorded from room temperature to 500 °C, at a heating rate of 10 °C min^−1^ and under an air flow rate of 30 mL min^−1^.

The application of CLCS as a green catalyst requires improvement in its thermal stability; thus, the incorporation of MWCNTs into its matrices is required. Thermogravimetric analysis (TG) is one of the most efficient techniques to elucidate the chemical and physical changes that occur in the polymer after its modification. The thermo-oxidative stability and degradability manner of the investigated CLCS and CLCS/MWCNTs composite were investigated using TG from room temperature to 500 °C at 10 °C min^−1^ rate of heating under air flow rate of 30 mL min^−1^. The percentage weight losses of CLCS and CLCS/MWCNTs composite, in comparison to the parent chitosan, that have been ascertained from the measurements of their TG are represented in Figure 6 and summarized in Table 1. 

Chitosan, CLCS and CLCS/MWCNTs composite displayed a special comparable degradation manner including two distinctive stages, during which perceivable losses of weight were obtained.

The first stage for the loss of weight began from 50 °C to 250 °C, during which chitosan, CLCS and CLCS/MWCNTs composite lost 10.47%, 10.05% and 11.33%, respectively, based on their initial weights (Table 1). These losses in weight can be ascribed to the evaporation of the moisture from the samples. The water loss above 100 °C is due to the hydrogen bonds between the polar groups in these samples and water molecules. 

The second thermal degradation step was sharp and started at 270 °C. The weight losses of chitosan, CLCS and CLCS/MWCNTs composite at 500 °C were 65.46%, 57.95% and 53.29%, respectively (Table 1). This thermal event may be attributed to the thermal decomposition of these samples which is preceded by a complex reaction involving the dehydration of their saccharide moieties, the random splitting of their repeating units and the liberation and vaporization of the small molecular degradative products from these samples. Moreover, the observed weight losses of both CLCS and CLCS/MWCNTs composite may also be attributed to loss of water during the thermally induced cyclodehydration process of the hydrazide groups of the cross-linking linkages into 1,3,4-oxadiazole moieties. This is not a true degradation process but rather a thermo-chemical transformation process to more thermally stable oxadiazole-containing polymers [29,30]. The thermal stability of the investigated samples could be arranged in the following order: CLCS/MWCNTs composite > CLCS > chitosan. It can be noted that the CLCS/MWCNTs composite exhibited the maximum thermo-oxidative stability, in comparison with those of the other samples, as illustrated by its minimum loss of weight as well as by its maximum weight residues at any given temperature from 310 °C to 500 °C. The higher thermal stability of CLCS/MWCNTs composite than that of CLCS is due to the presence of incorporated MWCNTs. CLCS is more thermally stable than chitosan because it possesses highly thermal stable aminobenzhydrazide cross-linking linkages.

### 2.6. Synthesis of Benzil Bis-Thiazoles

**Scheme 1:**
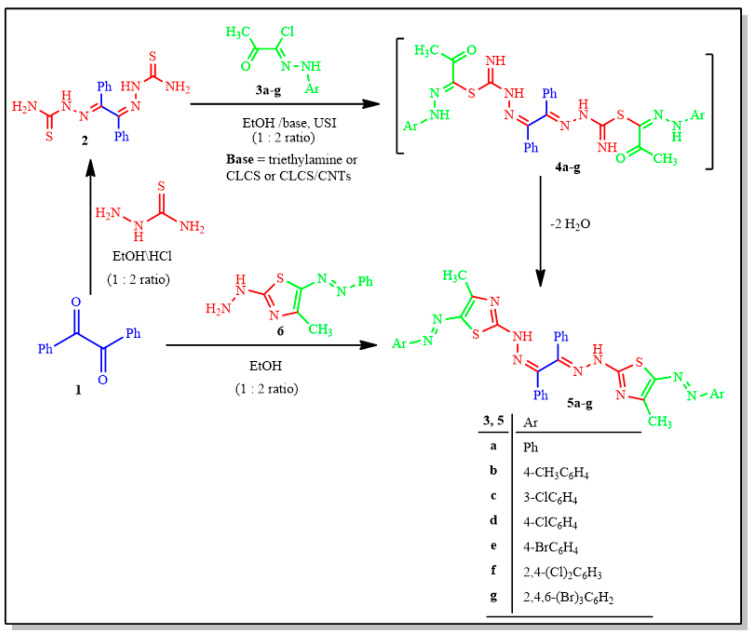
Synthesis of bis-thiazole derivatives **5a**–**g**.

Continuing our work on bis-heterocycle synthesis [31,32,33,34,35], the starting compound 2,2′-(1,2-diphenylethane-1,2-diylidene)bis(hydrazinecarbothioamide) **2** was obtained via reaction of benzil **1** with thiosemicarbazide in a molar ratio of 1:2 in refluxing EtOH/drops HCl for 3 h using a published procedure [36] (Scheme 1). 

The chemical reactivity of benzil bis-thiosemicarbazone **2** towards various hydrazonoyl chlorides **3** [37,38,39] was studied to prepare a new series of bis-thiazole derivatives. Thus, the reaction of compound **2** with *N*-aryl-2-oxopropanehydrazonoyl chlorides **3a**–**g** (two equivalents) in EtOH under ultrasonic irradiation (20–60 min) in the presence of TEA or the CLCS or CLCS/MWCNTs as basic catalysts afforded, after working up, bis-thiazoles **5a**–**g** (Scheme 1). The development of all reactions was tracked by thin layer chromatography (TLC). Identification of the best basic catalyst was initially examined (Table 2). 

As seen in Table 2, CLCS/MWCNT was the most effective of the ultrasonic basic catalysts. The reaction occurs efficiently with electron-deficient as well as electron-rich atoms or group of atoms for the phenyl group of hydrazonoyl halides **3**. We have observed that under the same reaction conditions the yields of the desired products **5a**–**g** increased by changing triethylamine into CLCS. Moreover, using CLCS/MWCNT as a basic catalyst has a significant increase effect on the product yields. 

The elemental analyses and spectroscopic data of the obtained products **5a**–**g** supported the assigned structures. The IR spectrum of **5a** as a representative example exhibits strong stretching frequencies in the region of 3379 cm^−1^, attributable to the NH group. Its ^1^H-NMR spectrum displayed two singlet signals for the 2CH_3_ and 2NH protons at *δ* 2.47 and 8.28 ppm, respectively, in addition to the characteristic multiplet signal for the twenty aromatic protons (10H × 2). The mass spectrum is additional evidence for supporting the obtained structure, which gave a molecular ion at *m*/*z* 640 [M]^+^. Furthermore, reaction of benzil with two equivalents of 2-hydrazinyl-4-methyl-5-(phenyl diazenyl) thiazole **6** [40] gave an identical product in IR, m.p. and mixed m.p. with **5a**, as depicted in Scheme 1.

**Scheme 2:**
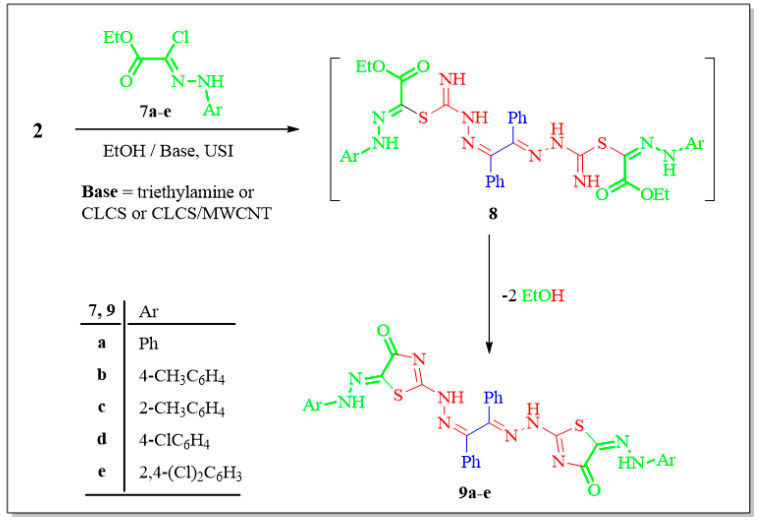
Synthesis of bis-thiazole derivatives **9a**–**e**.

Similarly, as shown in Scheme 2, treatment of compound **2** with ethyl 2-chloro-2-(2-arylhydrazono)acetates **7a**–**e** under the same reaction conditions was performed smoothly and produced the respective benzil bis-thiazolones **9a**–**e** as the final products. In addition, the effect of the basic catalyst type, such as TEA or the CLCS or CLCS/MWCNTs, on the yield % of the obtained products **9a**–**e** was examined (Table 3). 

Table 3 shows that the reaction progressed efficiently with different substituents of the phenyl group of hydrazonoyl chlorides **7a**–**e**. CLCS/MWCNTs were also more convenient basic catalysts for ultrasonic irradiation than TEA.

The structures of products **9a**–**e** were elucidated based on spectral and analytical data, as illustrated in the experimental section. For example, the IR spectra of the isolated products **9a**–**e** revealed the existence of the characteristic bands for the –NH and C=O groups at the normal wave numbers. The ^1^H-NMR spectra of compounds **9a**–**e** showed the expected two singlet signals corresponding to the two NH hydrazones, together with the expected aromatic protons.

## 3. Experimental Section

### 3.1. Measurements

The details of instruments used for characterization of the synthesized compounds are attached in the supplementary file.

### 3.2. Methods

#### 3.2.1. Preparation of CLCS

Benzaldehyde (20 mL) was gradually added to chitosan (5 g) swollen in methanol (60 mL) with continuous stirring at 25 °C overnight, then filtered and rinsed with methanol and dried. The obtained material (4 g) was stirred in NaOH solution (120 mL, 1 mmol/L) at 25 °C, then epichlorohydrin (6 mL) was added and the stirring was continued for six hours, before being filtered, rinsed with water then acetone and dried. To the resulting material (3 g), suspended in NaOH solution (90 mL, 1 mmol/L), a solution of 4-aminobenzhydrazide (2.19 g in 25 mL of DMF) was added, then stirred at 25 °C overnight, filtered, washed with methanol and dried. The produced material (2 g) was stirred in solution of ethanolic HCl (0.24 mol/L HCl) at 25 °C overnight, filtered, rinsed with ethanol and dried to obtain CLCS (Figure 1). This preparation method was described in detail in our recently published work [28].

#### 3.2.2. Preparation of CLCS/MWCNTs Composite

A total of 1 mg of MWCNTs (Nano Amour, New Mexico, USA, purity: >98%, outside diameter: 30–80 nm, inside diameter: 5–15 nm, length: <10 µm) was well dispersed in distilled water (50 mL) using a probe sonicator for an hour and then poured slowly into CLCS (0.2 g) swollen in acetic acid (150 mL, 1% *v*/*v*). The weight ratio of the MWCNTs: CLCS was 5 × 10^−3^ as reported in our previous work [28]. 

The resulting mixture was placed on a mechanical stirrer (850 rpm) at room temperature overnight for reaching an optimum homogeneity of MWCNTs dispersion inside the matrices of the CLCS. The resulting mixture was treated with sodium carbonate solution (1% *wt*/*v*) until acetic acid medium was fully neutralized, then filtered, washed repeatedly with distilled water, immersed into methanol and stirred overnight for dewatering and desalting. The CLCS/MWCNTs composite was filtered, rinsed with methanol and dried at 60 °C to constant weight (Figure 2). Formation of this nanocomposite was based on the fact that the pi-bonds of sidewalls of MWCNTs ionically interacted with the functional groups of the CLCS. A detailed description of this preparation procedure was given in our previous work [28]. 

#### 3.2.3. Synthesis of Benzil Bis-Thiazole Derivatives **5a**–**g** and **9a**–**e**

Method A: A mixture of 2,2′-(1,2-diphenylethane-1,2-diylidene)bis (hydrazinecarbothioamide) (**2**) (0.356 g, 1 mmol) and two equivalents of the proper hydrazonoyl chlorides **3a**–**g** or **7a**–**e** (2 mmol) in ethanol (20 mL) containing TEA (0.2 g, 2 mmol) was irradiated for 20–60 min with an ultrasonic generator at 50 °C. Radiation exposure continued until all the starting materials vanished and the product was developed; TLC supervised. The obtained precipitate of TEA/HCl was filtered off, and the mother liquor was evaporated. The formed solid product in each case was filtered off and finally crystallized from the appropriate solvent to give the respective bis-thiazole derivatives **5** or **9**. 

Method B: A mixture of **2** (0.356 g, 1 mmol) and two equivalents of **3a**–**g** or **7a**–**e** (2 mmol) containing CLCS (0.1 g) was irradiated for 20–40 min with an ultrasonic generator at 50 °C. To extract CLCS, the hot solution was filtered off and extra solvent was discarded under minimized pressure. The mixture was moistened with methanol and the precipitate was filtered, washed with methanol and finally recrystallized from EtOH or DMF to give derivatives of the respective products bis-thiazole **5** or **9**.

Method C: Same procedure as method B using CLCS/MWCNTs (0.1 g) instead of CLCS.

The physical constants and analytical data of synthesized products **5a**–**g** and **9a**–**e** are attached in the supplementary file. 

#### 3.2.4. Alternative Synthesis of **5a**

A solution of 10 mL 2-propanol containing benzil **1** (0.210 g, l mmol) and hydrazine derivative **6** (0.466 g, 2 mmol) was heated under reflux for 2 h. The formed precipitate was isolated via filtration then recrystallized from DMF to give product **5a** in a 71% yield.

## 4. Conclusions

In this article, a CLCS/MWCNTs composite was formulated and utilized as a reusable green nanocomposite heterogeneous biocatalyst and effectively used in a comparative study with TEA as a traditional catalyst for the synthesis of a novel series of benzil bis-thiazoles from the reaction of benzil bis-thiosemicarbazone with various hydrazonoyl chlorides under ultrasonic irradiation. In addition to the preferential green impact, the results obtained showed that the composite of CLCS/MWCNTs was a more powerful heterogeneous basic catalyst in those reactions compared to TEA. Additionally, their yield percentages were calculated using triethylamine (as a traditional catalyst) and CLCS/MWCNTs nanocomposite (as a green recyclable catalyst) in a comparative study. The reported catalyst is inexpensive for good yields of the bis-thiazoles and may be used in industrial production for the reported compounds.

## Figures and Tables

**Table 1:** Thermogravimetric analysis results of chitosan, CLCS and CLCS/MWCNTs composite at 10 °C min^−1^ heating rate in air.

Temperature (°C)	Weight Loss (%)		
	Chitosan	CLCS	CLCS/MWCNTs
50	2.24	0.48	0.72
100	6.36	1.92	3.62
150	8.61	4.79	6.75
200	8.98	7.42	8.44
250	10.47	10.05	11.33
260	11.22	11.72	12.54
270	13.09	15.55	14.47
280	15.33	22.26	17.60
290	19.08	29.20	21.95
300	25.06	34.94	27.48
310	34.04	37.81	32.55
320	40.76	39.97	35.68
330	43.76	41.64	37.86
340	46.01	43.07	39.79
350	47.87	44.51	41.23
400	53.86	48.93	46.53
450	59.09	53.20	50.15
500	65.46	57.95	53.29

**Table 2:** Comparison of synthesis of bis-thiazoles **5a**–**g** under ultrasound irradiation using a different basic catalyst on the time of reaction and the yield % of the products.

Compound no.	TEA		CLCS		CLCS/MWCNTs	
	Time (Min)	(%) Yield	Time (min)	(%) Yield	Time (min)	(%) Yield
5a	50	77	41	79	20	85
5b	50	74	43	80	23	86
5c	56	78	46	79	25	86
5d	53	72	43	80	28	84
5e	52	72	36	81	19	87
5f	60	78	38	83	25	85
5g	55	73	38	82	22	87

**Table 3:** Comparison of synthesis of bis-thiazoles **9a**–**e** under ultrasound irradiation using a different basic catalyst according to the time of reaction and the yield % of the products.

Compound no.	TEA		CLCS		CLCS/MWCNTs	
	Time (min)	(%) Yield	Time (min)	(%) Yield	Time (min)	(%) Yield
9a	53	72	39	77	22	86
9b	48	70	40	78	20	83
9c	53	72	44	80	23	84
9d	47	74	41	82	24	85
9e	55	74	38	80	21	83

## Data Availability

The data presented in this study are available on request from the corresponding author.

## Supplementary Materials

The following are available online at https://www.mdpi.com/article/10.3390/polym13111728/s1. Reference [29] has been cited in the supplementary file.
