# Supplementary material for: Cross-Linked Chitosan/Multi-Walled Carbon Nanotubes Composite as Ecofriendly Biocatalyst for Synthesis of Some Novel Benzil Bis-Thiazoles
Source: Polymers (Basel). 2021 May 25;13(11):1728. doi: 10.3390/polym13111728 (PMC8198799; doi:10.3390/polym13111728)
Supplement: Supplementary file 1 [file polymers-13-01728-s001.zip › polymers-1199435-supplementary.pdf]

polymers

Supplementary Materials

Cross-Linked Chitosan/Multi-walled Carbon Nanotubes Composite as Ecofriendly Biocatalyst for Synthesis of Some Novel Benzil Bis-Thiazoles

Latifah A. Alshabanah [1], Sobhi M. Gomha [2,3,*], Laila A. Al-Mutabagani [1,*], Tariq Z. Abolibda [3], Nahed A. Abd El-Ghany [2], Waleed A. M. A. El-Enany [2], Ahmed K. El-Ziaty [4], Rania S. Ali [5] and Nadia A. Mohamed [2,6]

1 Department of Chemistry, College of Science, Princess Nourah bint Abdulrahman University, Riyadh 11671, Saudi Arabia, laalsabanah@pnu.eud.sa (L.A.A.)
2 Department of Chemistry, Faculty of Science, Cairo University, Giza 12613, Egypt; smgomha@iu.edu.sa (S.M.G.); abdelghanyn@sci.cu.edu.eg (N.A.A.E.-G.); waleed_org_chem@yahoo.com (W.A.M.A.E.-E.); namadm@hotmail.com (N.A.M.)
3 Department of Chemistry, Faculty of Science, Islamic University in Almadinah Almonawara, Almadinah Almonawara 42351, Saudi Arabia; t.z.a@iu.edu.sa
4 Chemistry Department, Faculty of Science, Ain Shams University, El-Khalifa El-Mamoun, 11566 Abbassia, Cairo, Egypt; ahm512@sci.asu.edu.eg
5 Department of Basic Science, Faculty of Industrial Education Helwane University, Cairo 11795, Egypt; raniayousif2016@yahoo.com
6 Department of Chemistry, College of Science & Arts, Qassim University, Buraidah 51452. Saudi Arabia
* Correspondence: s.m.gomha@gmail.com (S.M.G.); laalmutbagani@pnu.edu.sa (L.A.A.-M.)

Citation: Alshabanah, L.A.; Gomha, S.M.; Al-Mutabagani, L.A.; Abolibda, T.Z.; Abd El-Ghany, N.A.; El-Enany, W.A.M.A., El-Ziaty, A.K.; Ali, R.S.; Mohamed, N.A. Cross-Linked Chitosan/ Multi-walled Carbon Nanotubes Composite as Ecofriendly Biocatalyst for Synthesis of Some Novel Benzil Bis-Thiazoles. *Polymers* **2021**, *13*, 1728. https://doi.org/10.3390/polym13111728

Academic editor: Łukasz Klapiszewski

Received: 11 April 2021
Accepted: 22 May 2021
Published: 25 May 2021

Publisher's Note: MDPI stays neutral with regard to jurisdictional claims in published maps and institutional affiliations.

Copyright: © 2021 by the authors. Licensee MDPI, Basel, Switzerland. This article is an open access article distributed under the terms and conditions of the Creative Commons Attribution (CC BY) license (http://creativecommons.org/licenses/by/4.0/).

3. Experimental section

3.1. Measurements

Melting points were determined on a digital melting point apparatus of the Electrothermal IA 9000 series. Elemental analyses were measured by using a German made Elementarvario LIII CHNS analyzer. Mass spectra were recorded at 70 eV on a mass spectrometer of the Shimadzu GCMS-QP1000 EX. NMR spectra were recorded on a Varian Mercury VX-300 NMR spectrometer operating at 300 MHz (^{1}H-NMR) and run in deuterated dimethylsulfoxide (DMSO-d6). Chemical shifts were related to that of the solvent. ^{13}C-NMR was recorded on a BRUKER spectrometer at 75 MHz. IR spectra were recorded in potassium bromide discs on Pye Unicam SP 3300 and Shimadzu FTIR 8101 PC infrared spectrophotometers. Surface morphology was investigated using scanning electron microscope (SEM), model Quanta FEG 250. Brucker's D-8 advanced wide-angle X-ray diffractiometer was used to investigate the inner structures of chitosan, CLCS, CLCS/MWCNTs composite and MWCNTs. Shimadzu TGA-50 H Thermal Analyzer was utilized to study the thermal degradation behavior of the samples from room temperature to 500 °C with a heating rate of 10 °C min^{-1} and under air flow rate of 30 mL min^{-1}.

3.2. Methods

3.2.1. Preparation of CLCS

Benzaldehyde (20 mL) was gradually added to chitosan (5 g) swollen in methanol (60 mL) with a continuous stirring at 25 °C overnight, then filtered and rinsed with methanol and dried. The obtained material (4 g) was stirred in NaOH solution (120 mL, 1 mmol/L) at 25 °C, then epichlorohydrin (6 mL) was added and the stirring was continued for six hours, filtered, rinsed with water then acetone and dried. To the resulted material (3 g) suspended in NaOH solution (90 mL, 1 mmol/L), a solution of 4-aminobenzhydrazide (2.19 g in 25 mL of DMF) was added, then stirred at 25 °C overnight, filtered, washed with methanol and dried. The produced material (2 g) was

stirred in solution of ethanolic HCl (0.24 mol/L HCl) at 25 °C overnight, filtered, rinsed with ethanol and dried to obtain CLCS (Chart 1). This preparation method was described in detail in our recently published work [29].

3.2.2. Preparation of CLCS/MWCNTs Composite

1 mg of MWCNTs (Nano Amour, USA, purity: > 98%, outside diameter: 30–80 nm, inside diameter: 5–15 nm, length: < 10 μm) was well dispersed in distilled water (50 mL) using a probe sonicator for an hour and then poured slowly into CLCS (0.2 g) swollen in acetic acid (150 mL, 1% *v*/*v*). The weight ratio of the MWCNTs: CLCS was 5×10^{-3} as reported in our previous work [29].

The resulted mixture was placed on a mechanical stirrer (850 rpm) at room temperature overnight for reaching an optimum homogeneity of MWCNTs dispersion inside the matrices of the CLCS. The resulting mixture was treated with sodium carbonate solution (1% *wt*/*v*), till acetic acid medium was fully neutralized, filtered, washed repeatedly with distilled water, immersed into methanol with stirring overnight for dewatering and desalting. The CLCS/MWCNTs composite was filtered, rinsed with methanol and dried in at 60 °C to constant weight (Figure 2). Formation of this nanocomposite was based on that the pi-bonds of sidewalls of MWCNTs were ionically interacted with the functional groups of the CLCS. A detailing description of this preparation procedure was given in our previous work [29].

3.2.3. Synthesis of Benzil Bis-Thiazole Derivatives 5a-g and 9a-e.

Method A:

A mixture of 2,2'-(1,2-diphenylethane-1,2-diylidene)bis(hydrazinecarbothioamide) (**2**) (0.356 g, 1 mmol) and two equivalents of the proper hydrazonoyl chlorides **3a-g** or **7a-e** (2 mmol) in ethanol (20 mL) containing TEA (0.2 g, 2 mmol) was irradiated 20–60 min with an ultrasonic generator at 50 °C. (Radiation exposure continued until all the starting materials vanished and the product was developed, TLC supervised). The obtained precipitate of TEA/HCl was filtered off, and the mother liquor was evaporated. The formed solid product in each case, was filtered off and finally crystallized from the appropriate solvent to give the respective bis-thiazole derivatives **5** or **9**.

Method B:

A mixture of **2** (0.356 g, 1 mmol) and two equivalents of **3a-g** or **7a-e** (2 mmol) containing CLCS (0.1 g) was irradiated 20–40 min with an ultrasonic generator at 50 °C. To extract CLCS, the hot solution was filtered off, and extra solvent was discarded under minimized pressure. The mixture was moistened with methanol and the precipitate was filtered, washed with methanol, and finally recrystallized from EtOH or DMF to give derivatives of the respective products bis-thiazole **5** or **9**.

Method C:

Same procedure in method B using CLCS/MWCNTs (0.1 g) instead of CLCS.

The physical constants and analytical data of synthesized products **5a-g** and **9a-e** are attached in the supplementary file.

1,2-Bis(2-(4-methyl-5-(phenyldiazenyl)thiazol-2-yl)hydrazono)-1,2-diphenylethane (5a). Red solid; m.p. 161–162 °C (EtOH); IR (KBr): *v* 3379 (NH), 3044, 3025 (C–H), 1601 (C=N) cm^{-1}; ^{1}H–NMR (DMSO-d_6): δ 2.47 (s, 6H, $2CH_3$), 7.02–7.76 (m, 20H, Ar–H), 8.28 (br s, 2H, 2NH, D_2O–exchangeable) ppm; ^{13}C–NMR (DMSO-d_6): δ 13.85 (CH_3), 115.08, 117.58, 119.80, 123.14, 126.77, 129.80, 130.21, 135.77, 137.23, 143.12, 149.14, 156.61(Ar–C and C=N) ppm; MS *m*/*z* (%): 640 (M^+, 41). Anal. Calcd. for $C_{34}H_{28}N_{10}S_2$ (640.78): C, 63.73; H, 4.40; N, 21.86. Found C, 63.70; H, 4.36; N, 21.75%.

1,2-Bis(2-(4-methyl-5-(p-tolyldiazenyl)thiazol-2-yl)hydrazono)-1,2-diphenylethane (5b). Red solid; m.p. 155–157 °C (EtOH); IR (KBr): *v* 3384 (NH), 3047, 3029 (C–H), 1600 (C=N) cm^{-1}; ^{1}H-NMR (DMSO-d_6): δ 2.27 (s, 6H, $2CH_3$), 2.41 (s, 6H, $2CH_3$), 6.89–7.82 (m, 18H, Ar–H), 7.95 (br s, 2H, 2NH, D_2O-exchangeable) ppm; MS *m/z* (%): 668 (M^+, 15). Anal. Calcd. for $C_{36}H_{32}N_{10}S_2$ (668.84): C, 64.65; H, 4.82; N, 20.94. Found C, 64.53; H, 4.72; N, 20.74%.

1,2-Bis(2-(5-((3-chlorophenyl)diazenyl)-4-methylthiazol-2-yl)hydrazono)-1,2-diphenylethane (5c). Red solid; m.p. 144–146°C (EtOH); IR (KBr): *v* 3383 (NH), 3052, 3024 (C–H), 1599 (C=N) cm^{-1}; ^{1}H-NMR (DMSO-d_6): δ 2.44 (s, 6H, $2CH_3$), 6.91–7.49 (m, 16H, Ar–H), 7.74 (s, 2H, Ar–H), 8.06 (br s, 2H, 2NH, D_2O-exchangeable) ppm; MS *m/z* (%): 711 (M^++2, 18), 709 (M^+, 7). Anal. Calcd. For $C_{34}H_{26}Cl_2N_{10}S_2$ (709.67): C, 57.54; H, 3.69; N, 19.74. Found C, 57.41; H, 3.55; N, 19.61%.

1,2-Bis(2-(5-((4-chlorophenyl)diazenyl)-4-methylthiazol-2-yl)hydrazono)-1,2-diphenylethane (5d). Red solid (86%); m.p. 169–171°C (EtOH); IR (KBr): *v* 3352 (NH), 3048, 3029 (C–H), 1600 (C=N) cm^{-1}; ^{1}H-NMR (DMSO-d_6): δ 2.42 (s, 6H, $2CH_3$), 6.93–7.72 (m, 18H, Ar–H), 8.01 (br s, 2H, 2NH, D_2O-exchangeable) ppm; MS *m/z* (%): 711 (M^++2, 48), 709 (M^+, 10). Anal. Calcd. For $C_{34}H_{26}Cl_2N_{10}S_2$ (709.67): C, 57.54; H, 3.69; N, 19.74. Found C, 57.63; H, 3.59; N, 19.72%.

1,2-Bis(2-(5-((4-bromophenyl)diazenyl)-4-methylthiazol-2-yl)hydrazono)-1,2-diphenylethane (5e). Red solid, m.p. 161–163°C (EtOH); IR (KBr): *v* 3390 (NH), 3047, 3028 (C–H), 1601 (C=N) cm^{-1}; ^{1}H-NMR (DMSO-d_6): δ 2.45 (s, 6H, $2CH_3$), 6.86–7.75 (m, 18H, Ar–H), 8.00 (br s, 2H, 2NH, D_2O-exchangeable) ppm; MS *m/z* (%): 758 (M^++2, 17), 756 (M^+, 14). Anal. Calcd. for $C_{34}H_{26}Br_2N_{10}S_2$ (756.45): C, 51.14; H, 3.28; N, 17.54. Found C, 51.04; H, 3.17; N, 17.42%.

1,2-Bis(2-(5-((2,4-dichlorophenyl)diazenyl)-4-methylthiazol-2-yl)hydrazono)-1,2-diphenylethane (5f). Red solid, m.p. 154–156°C (EtOH); IR (KBr): *v* 3372 (NH), 3053, 3024 (C–H), 1603 (C=N) cm^{-1}; ^{1}H-NMR (DMSO-d_6): δ 2.45 (s, 6H, $2CH_3$), 7.35–7.81 (m, 16H, Ar–H), 8.15 (br s, 2H, 2NH, D_2O-exchangeable) ppm; MS *m/z* (%): 778 (M^+, 18). Anal. Calcd. for $C_{34}H_{24}Cl_4N_{10}S_2$ (778.56): C, 52.45; H, 3.11; N, 17.99. Found C, 52.26; H, 3.04; N, 17.86%.

1,2-Bis(2-(4-methyl-5-((2,4,6-tribromophenyl)diazenyl)thiazol-2-yl)hydrazono)-1,2-diphenylethane (5g). Red solid; m.p. 196–198°C (DMF); IR (KBr): *v* 3377 (NH), 3055, 3023 (C–H), 1600 (C=N) cm^{-1}; ^{1}H-NMR (DMSO-d_6): δ 2.41 (s, 6H, $2CH_3$), 7.40–8.19 (m, 10H, Ar–H), 8.37 (s, 4H, Ar–H), 8.65 (br s, 2H, 2NH, D_2O-exchangeable) ppm. Anal. Calcd. for $C_{34}H_{22}Br_6N_{10}S_2$ (1114.16): C, 36.65; H, 1.99; N, 12.57. Found C, 36.69; H, 1.85; N, 12.46%.

2,2'-(2,2'-(1,2-Diphenylethane-1,2-diylidene)bis(hydrazin-1-yl-2-ylidene))bis(5-(2-phenylhydrazono)thiazol-4(5*H*)-one) (9a). Yellow solid; m.p. 151–153°C (EtOH); IR (KBr): *v* 3393, 3260 (2NH), 3060, 2925 (C–H), 1673 (C=O), 1602 (C=N) cm^{-1}; ^{1}H-NMR (DMSO-d_6): δ 6.90–7.77 (m, 20H, Ar–H), 8.83 (br s, 2H, 2NH, D_2O-exchangeable), 10.49 (br s, 2H, 2NH, D_2O-exchangeable) ppm; MS *m/z* (%): 644 (M^+, 37). Anal. Calcd. for $C_{32}H_{24}N_{10}O_2S_2$ (644.73): C, 59.61; H, 3.75; N, 21.72. Found C, 59.53; H, 3.70; N, 21.58%.

2,2'-(2,2'-(1,2-Diphenylethane-1,2-diylidene)bis(hydrazin-1-yl-2-ylidene))bis(5-(2-(p-tolyl)hydrazono)thiazol-4(5*H*)-one) (9b).Yellow solid; m.p. 140–142°C (EtOH); IR (KBr): *v* 3406, 3259 (2NH), 3052, 2928 (C–H), 1675 (C=O), 1608 (C=N) cm^{-1}; ^{1}H-NMR (DMSO-d_6): δ 2.25 (s, 6H, $2CH_3$), 6.90–7.70 (m, 18H, Ar–H), 7.87 (br s, 2H, 2NH, D_2O-exchangeable), 10.55 (br s, 2H, 2NH, D_2O-exchangeable) ppm; ^{13}C-NMR (DMSO-d_6): δ 21.10 (CH_3), 122.88, 127.53, 130.07, 130.61, 131.43, 133.78, 145.25, 147.32, 154.23, 159.54, 162.10 (Ar–C and C=N), 175.76 (C=O) ppm; MS *m/z* (%): 672 (M^+, 26). Anal. Calcd. for $C_{34}H_{28}N_{10}O_2S_2$ (672.78): C, 60.70; H, 4.19; N, 20.82. Found C, 60.62; H, 4.04; N, 20.69%.

2,2'-(2,2'-(1,2-Diphenylethane-1,2-diylidene)bis(hydrazin-1-yl-2-ylidene))bis(5-(2-(o-tolyl)hydrazono)thiazol-4(5*H*)-one) (9c). Yellow solid; m.p. 135–137°C (EtOH); IR (KBr): *v* 3374, 3261 (2NH), 3057, 2936 (C–H), 1669 (C=O), 1598 (C=N) cm^{-1}; ^{1}H-NMR (DMSO-d_6): δ 2.35 (s, 6H, $2CH_3$), 6.87–7.75 (m, 18H, Ar–H), 7.83 (br s, 2H, 2NH, D_2O-exchangeable), 10.41 (br s, 2H, 2NH, D_2O-exchangeable) ppm; MS *m/z* (%): 672 (M^+, 15). Anal.

Calcd. for $C_{34}H_{28}N_{10}O_2S_2$ (672.78): C, 60.70; H, 4.19; N, 20.82. Found C, 60.54; H, 4.11; N, 20.70%.

2,2'-(2,2'-(1,2-Diphenylethane-1,2-diylidene)bis(hydrazin-1-yl-2-ylidene))bis(5-(2-(4-chlorophenyl)hydrazono)thiazol-4(5*H*)-one) (9d). Yellow solid; m.p. 157–159 °C (EtOH); IR (KBr): *v* 3388, 3252 (2NH), 3064, 2929 (C–H), 1687 (C=O), 1604 (C=N) cm^{-1}; ^1H-NMR (DMSO-d_6): δ 6.91–7.46 (m, 18H, Ar–H), 7.99 (br s, 2H, 2NH, D_2O-exchangeable), 10.69 (br s, 2H, 2NH, D_2O-exchangeable) ppm; MS *m/z* (%): 715 (M^{+}+2, 40), 713 (M^+, 11). Anal. Calcd. for $C_{32}H_{22}Cl_2N_{10}O_2S_2$ (713.62): C, 53.86; H, 3.11; N, 19.63. Found C, 53.77; H, 3.05; N, 19.47%.

2,2'-(2,2'-(1,2-Diphenylethane-1,2-diylidene)bis(hydrazin-1-yl-2-ylidene))bis(5-(2-(2,4-dichlorophenyl)hydrazono)thiazol-4(5*H*)-one) (9e). Yellow solid; m.p. 166–168 °C (EtOH); IR (KBr): *v* 3405, 3227 (2NH), 3048, 2919 (C-H), 1690 (C=O), 1600 (C=N) cm^{-1};^1H-NMR (DMSO-d_6): δ 7.11–7.78 (m, 16H, Ar-H), 8.17 (br s, 2H, 2NH, D_2O-exchangeable), 10.12 (br s, 2H, 2NH, D_2O-exchangeable) ppm; MS *m/z* (%): 782 (M^+, 11). Anal. Calcd. for $C_{32}H_{20}Cl_4N_{10}O_2S_2$ (782.51): C, 49.12; H, 2.58; N, 17.90. Found C, 49.04; H, 2.52; N, 17.77%.

Alternative synthesis of 5a.

A solution of 10 mL 2-propanol containing benzil **1** (0.210 g, l mmol) and hydrazine derivative **6** (0.466 g, 2 mmol) was heated under reflux for 2 h. The formed precipitate was isolated via filtration then recrystallized from DMF to give product **5a** in 71% yield**.**
